# Permeability of Buccal Mucosa

**DOI:** 10.3390/pharmaceutics13111814

**Published:** 2021-10-31

**Authors:** Apipa Wanasathop, Priya B Patel, Hyojin A. Choi, S. Kevin Li

**Affiliations:** Division of Pharmaceutical Sciences, College of Pharmacy, University of Cincinnati, 231 Albert Sabin Way, MSB # 3005, Cincinnati, OH 45267, USA; wanasaaa@mail.uc.edu (A.W.); patel5pi@mail.uc.edu (P.B.P.); choih4@mail.uc.edu (H.A.C.)

**Keywords:** permeability, buccal, membrane, drug delivery, transport

## Abstract

The buccal mucosa provides an alternative route of drug delivery that can be more beneficial compared to other administration routes. Although numerous studies and reviews have been published on buccal drug delivery, an extensive review of the permeability data is not available. Understanding the buccal mucosa barrier could provide insights into the approaches to effective drug delivery and optimization of dosage forms. This paper provides a review on the permeability of the buccal mucosa. The intrinsic permeability coefficients of porcine buccal mucosa were collected. Large variability was observed among the published permeability data. The permeability coefficients were then analyzed using a model involving parallel lipoidal and polar transport pathways. For the lipoidal pathway, a correlation was observed between the permeability coefficients and permeant octanol/water partition coefficients (*K_ow_*) and molecular weight (MW) in a subset of the permeability data under specific conditions. The permeability analysis suggested that the buccal permeation barrier was less lipophilic than octanol. For the polar pathway and macromolecules, a correlation was observed between the permeability coefficients and permeant MW. The hindered transport analysis suggested an effective pore radius of 1.5 to 3 nm for the buccal membrane barrier.

## 1. Introduction

For effective drug delivery across a biological membrane, it is important to understand the transport behavior of the membrane for the drugs. The mechanisms of drug permeation across a biological membrane can be divided into passive, facilitated, and active transport. The effects of the metabolic barrier due to enzyme degradation and drug clearance from the tissue due to blood circulation and the lymphatic system can also be important. For example, effective drug clearance after tissue penetration is beneficial to systemic drug delivery but has a negative impact on local drug delivery. In general, the barrier property of a membrane due to passive transport can be described by its permeability coefficient.

Previous reviews have covered a variety of topics in buccal drug delivery. These review papers, which span the last several decades, include general overviews and updates of buccal drug delivery and its assessments [1,2,3,4,5,6,7], drug products utilizing this route of administration [8,9], and technologies to improve drug delivery via the buccal mucosa such as penetration enhancers, microadhesives, nanoparticles, and biopolymers [10,11,12,13,14,15,16,17,18]. In addition, buccal delivery of specific drugs and their clinical uses have been reviewed [19,20,21,22]. Review papers are also available on the topics of buccal delivery of macromolecules such as peptides, oligonucleotides, and vaccines [7,23,24,25,26]. Consequently, these topics are not the focus of the present review paper.

For the permeability of buccal mucosa, previous studies have analyzed the relationships between the permeability coefficients and physicochemical properties of drugs [27,28]. The effect of temperature and activation energy of membrane permeation were also investigated to understand the transport mechanism of buccal mucosa [29]. However, a comprehensive summary of buccal permeability data is not available in the literature. The present paper provides a review on the permeability of the buccal mucosa for drug delivery. Porcine buccal mucosa has been the most common tissue in buccal drug delivery studies and was the focus of this review. The permeability coefficients of the buccal mucosa without the influence of formulations or the use of penetration enhancers (i.e., membrane intrinsic permeability) were summarized in this paper. The effects of lipophilicity and molecular weight of the permeants on their penetration across the buccal mucosa were examined. Analyses were performed to provide insights into a possible quantitative structure permeability relationship of passive transport across the buccal membrane that could be valuable in future drug delivery development.

## 2. Buccal Mucosa and Drug Delivery

Before the examination of buccal membrane permeability, this section provides a brief review of the buccal mucosa and buccal drug delivery. The oral mucosa is a complex series of tissues lining the oral cavity. It consists of tissue layers such as stratified squamous epithelium, basement membrane, and supporting connective tissues underneath. The buccal mucosa, in addition to the sublingual and gingival mucosa, is part of the oral mucosa. It is the area inside the cheek and between the gums and lower lips with an average surface area of ~100 cm^2^ [30]. The buccal mucosa consists of the outer epithelium and basement membrane. The non-keratinized stratified squamous epithelium forms the outer buccal epithelium. It is composed of mostly phospholipids and also proteins in the form of tonofilament. The basal layer of the epithelium differentiates into replacement cells that are shed from the outermost buccal surface. The epithelium, due to its morphology and lipid structure, is considered as the major barrier for the penetration of most drugs in buccal delivery. The basement membrane is a continuous layer of extracellular material and generally not considered as the major barrier for drug delivery. After the drug penetrates the buccal epithelium, it enters the systemic circulation via the vascularized tissue and jugular vein.

Buccal drug administration provides an alternative route for systemic drug delivery. It has several advantages over the gastrointestinal (GI) route by bypassing the hepatic first-pass effect, avoiding interference from acidity and enzymes relative to the GI tract, providing ease of dosing with the accessibility of the oral cavity and ease of drug removal in the event of adverse reactions. There are a number of commercial buccal drug delivery dosage forms in the market such as buccal tablet, spray, mucoadhesive, sublingual lozenge, chewing gum, film, and oromucosal solution. The disadvantages of buccal drug delivery include the (a) barrier of the buccal mucosa that its permeability may be too low for certain drugs, (b) interference of saliva that can dilute the drug for absorption, and (c) variable environment in the oral cavity due to food consumption and other daily activities. For example, although the permeability of the buccal mucosa is generally higher than that of the stratum corneum [31], it is generally lower than that of the GI mucosal monolayer [32,33], as can be seen by comparing their values to the buccal permeabilities presented later in the present review. To overcome this barrier, chemical penetration enhancers such as surfactants, physical penetration enhancers such as iontophoresis, and formulation technologies such as mucoadhesive and polymeric dosage forms can be utilized for buccal drug delivery. In order to fully utilize these enhancement methods and to develop effective buccal drug delivery systems, it is important to understand the intrinsic passive permeability of the buccal mucosa.

## 3. Permeability of Buccal Mucosa and Data Variability

The permeants and their buccal permeability coefficients collected for this review are listed in Table A1 (see Appendix A). The permeability coefficients were obtained from the references [27,28,29,34,35,36,37,38,39,40,41,42,43,44,45,46,47,48,49,50,51,52,53,54,55,56,57,58,59,60,61,62,63,64,65,66,67,68,69,70,71,72,73,74,75,76,77,78,79,80,81,82,83,84,85,86,87,88,89,90,91,92,93,94,95,96,97,98,99,100,101,102,103,104,105,106,107] and the logarithmic values of the permeability coefficients in cm/s (log *P*) were calculated. Only permeability data from porcine buccal mucosa were collected for the analysis because the majority of previous permeation studies was with the porcine tissue. In addition, a comparison between the porcine tissue and tissues from other species (bovine, canine, hamster, monkey, and human) showed that their permeability coefficients were within similar orders of magnitudes except for hamster tissue that displayed higher permeability (Table 1). In some cases, engineered tissues (TR146 tissue model, EpiOral tissue model, and Caco-2 cells) also showed higher permeability than the porcine buccal tissue. It should be noted that the data shown in Table 1 are not intended to be comprehensive but to support the use of porcine buccal permeability data in the analyses of the present review. In the data collection, another selection criterion was unperturbed buccal membrane. Only buccal permeability coefficients (intrinsic permeability) without formulation influences and organic solvent manipulations were considered. Results obtained at temperatures outside the normal range of 34–37 °C were not included. For the model analyses, the physicochemical properties of the permeants such as pKa, octanol/water partition coefficient (*K_ow_*), and octanol/water distribution coefficient (*D_ow_*), when available, were also collected from the previous studies (Table A1). *D_ow_*, which is the ratio of the concentration of unionized species in the octanol phase to the concentration of both ionized and unionized species in the aqueous phase, is related to *K_ow_* as described by Equation (A2) (see Appendix B). When the pKa, *K_ow_*, or *D_ow_* values were not available in the references, the pKa and *K_ow_* values were obtained from PubChem [108] and *D_ow_* was calculated using the pKa and pH of the permeant solutions in the studies (see Equations (A3) and (A4)).

**Figure 1:**
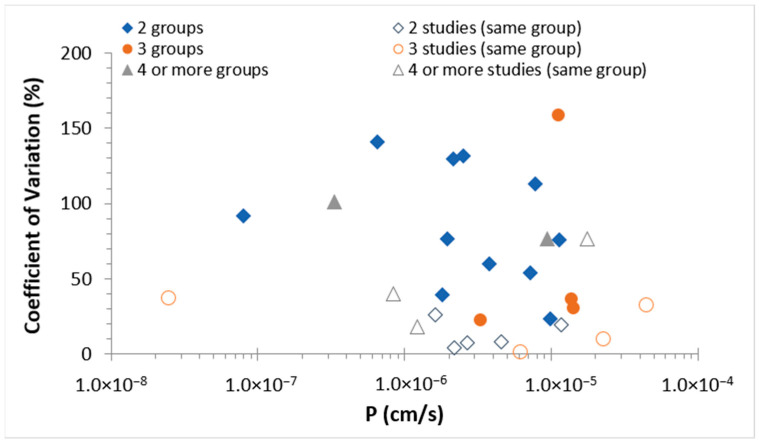
Assessment of permeation data variability. CV of the permeability data among different research groups were plotted against the average of the permeability data. In this case, CV was calculated using the reported mean values of the same permeant from different studies conducted by different research groups and plotted as a data point using the average of these values. CV of the data among different studies from the same research groups were also determined for comparison. In this case, CV was calculated using the reported mean values of the same permeant from different studies conducted by the same research group. Permeants in studies from different research groups are: antipyrine, atenolol, bupivacaine, buspirone, caffeine, didanosine, dideoxycytidine, estradiol, mannitol, metoprolol (2 data sets of different conditions), naltrexone, ondansetron, oxprenolol, propranolol (2 data sets of different conditions), and triamcinolone acetonide. Symbols: permeant data from two (closed diamonds), three (closed circles), and four or more (closed triangles) research groups. Permeants in studies from the same research groups are: acyclovir, antipyrine, caffeine, decitabine, diazepam, didanosine, estradiol, mannitol, morphine, nicotine (2 data sets of different conditions), triamcinolone acetonide. Symbols: permeant data from two (open diamonds), three (open circles), and four or more (open triangles) studies from the same research groups.

When permeability data of the same permeants are available from buccal permeation studies performed by multiple research groups under similar experimental conditions, large variability was observed between the data generated among these research groups. To compare the permeability data in these studies (from different research groups), the average of the mean permeability coefficients presented in these studies was determined for the permeant and the coefficient of variation (CV) was calculated (CV = ratio of the standard deviation to the average × 100%). The CV of the permeability coefficients was then plotted against the average permeability coefficient of each permeant (Figure 1). Although the high CV from the n = 2 studies can be related to the nature that only two studies were compared, most permeants have CV much higher than 50% even without considering the variability of the n = 2 studies. This is significantly higher than the CV generally encountered in diffusion cell permeation experiments [119]. Possible causes of the large variability could be the (a) different sources of porcine tissues (age, species), (b) different methods of tissue preparation and the resulting tissue conditions, and (c) different experimental conditions among these studies. For comparison, the average and CV of the mean permeability coefficients from studies of the same groups (from different permeation studies such as different papers published by the same research group) were also calculated and the CV was plotted against the average permeability coefficient of each permeant in the figure. The CV values from the same research groups are generally smaller than those from different research groups (open symbols vs. closed symbols, respectively). Furthermore, there is no apparent relationship between the variabilities and permeability coefficients of the permeants in both comparisons (of the same and different research groups), indicating that the variabilities were likely not transport-mechanism related. The large variabilities observed among the permeability data of the same permeants from the studies published by different research groups suggested the potential difficulty in using the literature permeability data to determine the quantitative structure permeability relationship for buccal drug delivery.

For the permeability analyses in the present review, when the permeability data were from different studies of the same research group, the average value of the permeability coefficients published in these studies was calculated and used as an individual data point. When the permeability data were from different research groups, the result from each research group was treated as an individual data point in the analyses; these data were analyzed as separate data points with the same weighing factor as if they were different permeants.

## 4. Membrane Transport Theory

In order to examine the relationships between the permeability coefficients of buccal mucosa and the physicochemical properties of the permeants, this section provides a brief review of transport theory for drug permeation across biological membranes. In general, the permeability of a biological membrane can be described by a parallel pathway permeation model of lipoidal and polar pathways [120,121]. The lipoidal pathway (or transcellular pathway) describes the permeation across the lipid barrier from the membrane lipid lamella structure, and the polar pathway (or pore pathway) describes the permeation across the aqueous channels across the membrane via the paracellular route or membrane defects. For the permeation of a weak acid or weak base, which is pH dependent, the flux of the permeant and its permeability coefficient can be expressed as [32]:

(1) $$J=J_{u}+J_{i}=\left(P_{u}C_{u}+P_{i}C_{i}\right)=\left(P_{u}f_{u}+P_{i}(1-f_{u})\right)C$$

(2) $$P=\left(P_{l}+P_{p}\right)f_{u}+P_{p}(1-f_{u})=P_{l}f_{u}+P_{p}$$

where *J* is flux, *P* is permeability coefficient of the membrane, *C* is concentration, *f_u_* is the fraction of unionized permeant, subscripts *u* and *i* represent the unionized and ionized permeants, and subscripts *l* and *p* represent the lipoidal and polar pathways, respectively. Assuming that the microenvironment of the lipid lamellae in the membrane can be mimicked by octanol and the contribution of *P_p_* is minimal, and using the relationship between membrane diffusion coefficient and permeant molecular weight [31], the permeability coefficient can be expressed as (see derivation of Equation (A10) in Appendix B):

(3) $$\log P=\log D_{ow}+c\;MW+constant$$

where *MW* is permeant molecular weight, *c* is the coefficient of *MW*, and *constant* is a constant. For permeants that are not affected by pH, *D_ow_* = *K_ow_* and Equation (3) becomes:

(4) $$\log P=\log K_{ow}+c\;MW+constant$$

Equations (3) and (4) were the models used in the analyses of the buccal permeability data (see Section 5).

When the microenvironment of the lipid barrier is different from that of octanol, membrane partitioning can be described by the linear free energy relationship [122]:

(5) $$\log K_{m}=a\log K_{ow}+\log b.\;$$

where *K_m_* is membrane partition coefficient and *a* and *b* are constants. The slope of the log *P* vs. log *K_ow_* relationship (or log *P* vs. log *D_ow_* for the permeants that are pH dependent due to ionization) indicates the lipophilicity of the rate limiting barrier for permeation across the membrane. Replacing *K_ow_* in the derivation of Equation (3) by this free energy relationship, Equation (4) can be rewritten as (see the derivation of Equation (A13) in Appendix B):

(6) $$\log(P/D_{ow})=\left(a-1\right)\log K_{ow}+c\;MW+constant$$

In addition to Equations (3) and (4), the buccal permeability data were also analyzed by Equation (6) (see Section 5).

For permeation across the polar pathway in a biological membrane, which can be modeled as aqueous channels with hindered transport, the permeability coefficient of the polar pathway (*P_p_*) can be described by the polar pathway transport model [123,124].

(7) $$P_{p}=\varepsilon K_{p}HD_{aq}/{h_{m}}^{\prime \prime }$$

where *ε* is membrane porosity, *K_p_* is the partition coefficient due to permeant-to-membrane interactions, *D_aq_* is aqueous diffusion coefficient, *H* is hindered transport factor for diffusion, and *h_m_″* is the effective thickness of the membrane for the polar pathway. The hindered transport factor is a function of the permeant molecular size and the pore size of the polar pathway [125].

(8) $$H=6\pi {\left(1-\lambda \right)}^{2}/\left[2.25\pi^{2}\sqrt{2}{\left(1-\lambda \right)}^{-2.5}\left(1+\sum_{n=1}^{2}a_{n}{\left(1-\lambda \right)}^{n}\right)+\sum_{0}^{4}a_{n+3}\lambda^{n}\right]$$

where λ is the ratio of permeant radius to pore radius, *a*_1_ = −1.21667, *a*_2_ = 1.53359, *a*_3_ = −22.5083, *a*_4_ = −5.6117, *a*_5_ = −0.3363, *a*_6_ = −1.216, and *a*_7_ = 1.647. When λ < 0.4, Equation (8) is equivalent to the commonly used Renkin equation. The hindered transport model can be used to characterize the effective size of a pore transport pathway [126,127,128]. Assuming that the effects due to permeant-to-membrane interactions in the aqueous transport pathway are small (i.e., *K_p_* ≈ 1), the ratio of the permeability coefficients of two permeants is related to the ratio of hindered transport factors of the permeants.

(9) $$\left(P_{p,1}/D_{aq,1}\right)/\left(P_{p,2}/D_{aq,2}\right)=H_{1}/H_{2}$$

where subscripts 1 and 2 represent permeant 1 and permeant 2, respectively. By fitting Equation (9) with the permeability ratio data, the effective pore radius of the polar pathway can be evaluated (see Section 6).

## 5. Effects of Lipophilicity and Molecular Weight on the Permeation of Small Molecules

**Figure 2:**
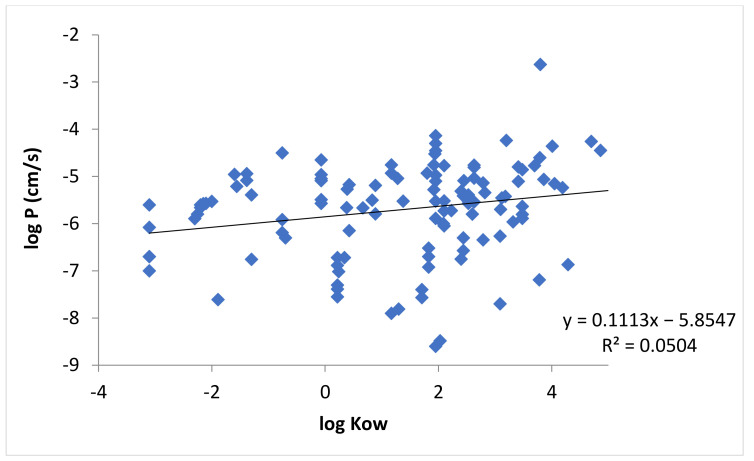
Relationship between log *P* and log *K_ow_* for all applicable permeants in Table A1.

**Figure 3:**
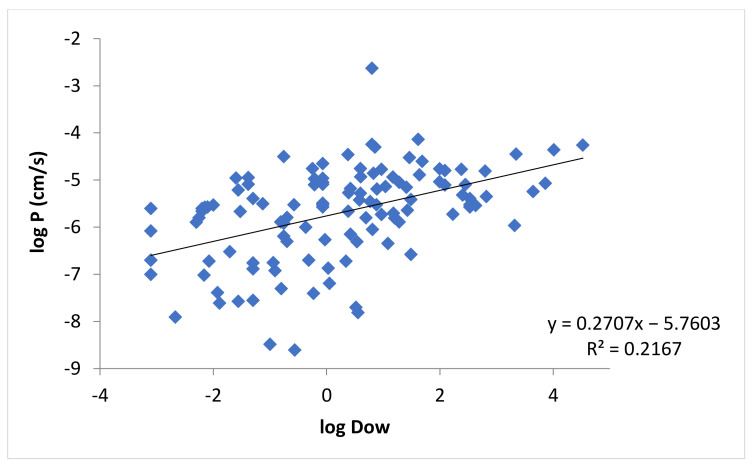
Relationship between log *P* and log *D_ow_* for all applicable permeants in Table A1.

Figure 2 shows the relationship between the permeability coefficients of buccal mucosa and the lipophilicities of the permeants in Table A1, in which the lipophilicities are measured by *K_ow_*. The correlation between log *P* and log *K_ow_* was poor. By taking into account the fraction of ionization of the permeants, Figure 3 shows the relationship between the permeability coefficients and *D_ow_* of the permeants. The correlation between the permeability coefficients and lipophilicities of the permeants improved when *D_ow_* (instead of *K_ow_*) was used (Figure 3 vs. Figure 2), but the correlation was still relatively poor.

**Figure 4:**
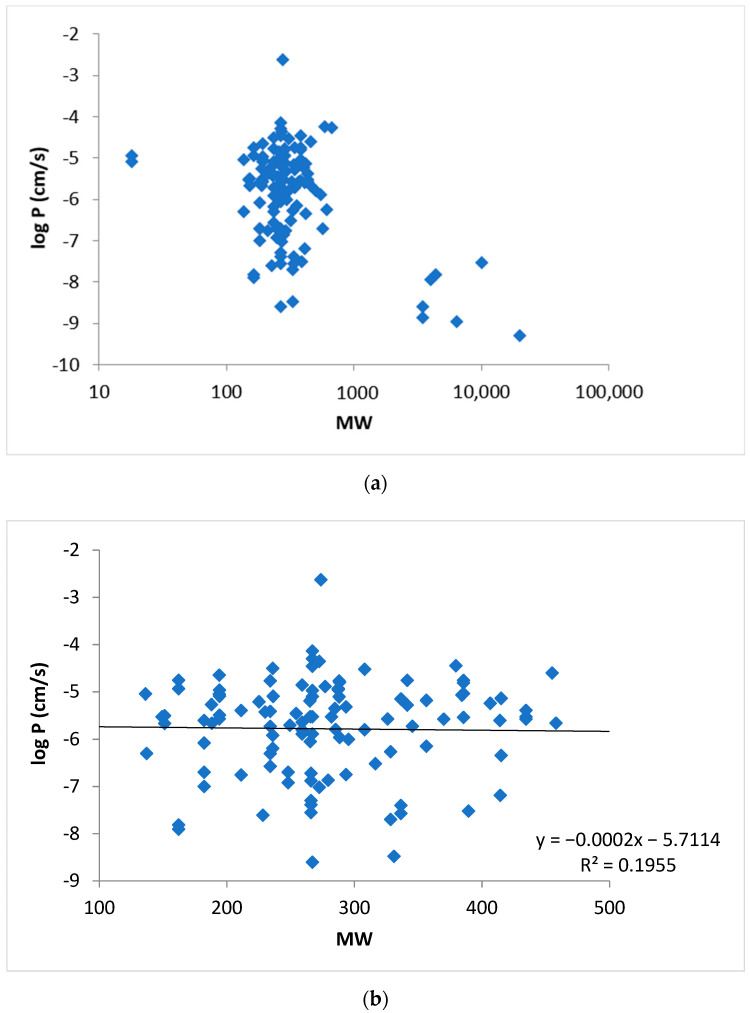
(**a**) Relationship between log *P* and *MW* for all applicable permeants in Table A1. (**b**) Enlarged figure to evaluate the relationship between log *P* and *MW* of permeants in the 130–460 Dalton range.

Figure 4 shows the relationship between the permeability coefficients of buccal mucosa and the MW of the permeants. There was no direct correlation between the permeability coefficients and MW of the permeants without the consideration of their lipophilicities. The effect of permeant MW on membrane permeability was then investigated by regression analyses (MS Excel Linest function) using log *D_ow_* and *MW* as two independent variables. Three parameters (*a*, *c*, and *constant*) were fitted to the experimental data using a similar relationship as Equation (3):

(10) $$\log P=a\log D_{ow}+c\;MW+constant$$

Table 2 presents the result of this analysis. The incorporation of *MW* in the permeability analysis did not have any significant effects on the correlation between log *P* and log *D_ow_* for the quantitative structure permeability relationship.

Equation (10) was derived under the assumption of minimal contribution from the polar pathway to the total permeability of the buccal membrane (i.e., *P_p_* << *P*; see the assumption in Equation (A8) in Appendix B). For example, permeants that have low *D_ow_* (e.g., *D_ow_* < −1) could predominantly utilize the polar pathway for buccal membrane permeation and have low or comparable permeability coefficients to those of the polar pathway, so the inclusion of these data in the analysis can introduce errors. To this end, log *D_ow_* < −1 was applied as an exclusion criterion in the evaluation of the “log *P* vs. log *D_ow_* and *MW*” relationship; only the permeability data of permeants with log *D_ow_* > −1 and water were analyzed. Water was not excluded in the analyses because it could readily permeate lipid bilayers without using the polar pathway. The exclusion of these data did not improve the “log *P* vs. log *D_ow_* and *MW*” correlation and further exclusion of the permeants that have *D_ow_* < 0 also did not show any improvement (Table 2). To account for possible differences between *K_m_* and *K_ow_*, additional analyses were performed using Equation (6) and the permeability data. The results are presented in Table 2. The approach of log (*P/D_ow_*) vs. log *K_ow_* and *MW* provided better correlations than those of the “log *P* vs. log *D_ow_* and *MW*” when compared using the same permeability datasets (first row vs. fourth row, second row vs. fifth row, and third row vs. sixth row in Table 2). However, except for the dataset of “all data” (fourth row in Table 2), the correlations were still relatively poor. The improvement in correlations using the model of Equation (6) is consistent with the conclusion that the buccal membrane barrier is less lipophilic than octanol (*K_m_* vs. *K_ow_*; see discussion later in this section and Section 7). For the correlation using all data, the quantitative structure permeability relationship observed was: log (*P/D_ow_*) = −0.56 log *K_ow_* −0.0014 *MW* −4.8 (with R^2^ = 0.588).

**Figure 5:**
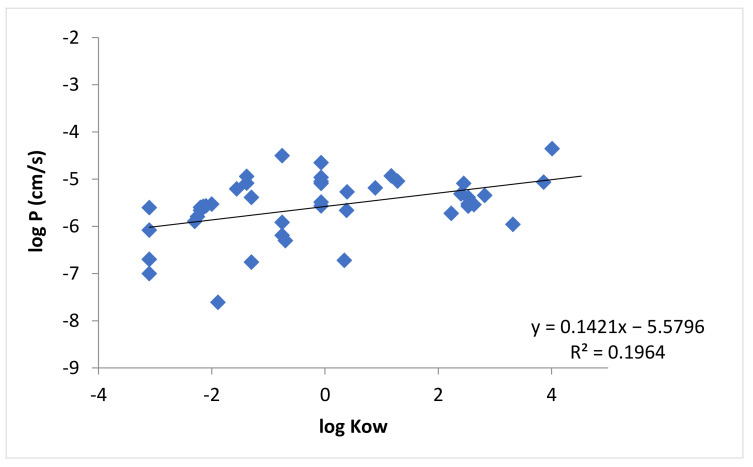
Relationship between log *P* and log *K_ow_* for permeants that are not affected by pH ionization (pH independent permeants) in Table A1.

Membrane transport of weak acid and weak base can be affected by a number of factors including the fraction of permeant ionization that is a function of permeant pKa and solution pH (i.e., pH dependent permeant charges). Due to this uncertainty, permeant transport that is pH dependent and is related to the fraction of permeant ionization can introduce errors in the permeability analyses. Figure 5 shows the relationship between the permeability coefficients and *K_ow_* by excluding the permeants with ionization as a function of pH (i.e., using only pH independent permeants) in the analysis. The exclusion of the pH dependent permeants slightly improved the correlation of the relationship between log *P* and log *K_ow_* (compared to Figure 2).

With the pH independent permeants as the dataset, regression analyses were performed using log *K_ow_* and *MW* as two independent variables, *a*, *c*, and *constant* as the parameters, and a similar relationship as Equation (4):

(11) $$\log P=a\log K_{ow}+c\;MW+constant$$

The incorporation of *MW* in the analysis improved the correlation (Table 3). To consider the influence of the polar pathway (*P_p_*), the exclusion criteria of log *K_ow_* < −1 and < 0 were applied to the analyses of these permeants. The exclusion of the permeants that have log *K_ow_* < −1 provided a better correlation of the “log *P* vs. log *K_ow_* and *MW*” relationship. Increasing the log *K_ow_* value in the exclusion criterion to analyze only permeants with log *K_ow_* > 0 further improved the “log *P* vs. log *K_ow_* and *MW*” correlation (R^2^ = 0.615). The results of these analyses are summarized in Table 3.

An interesting observation in the present study is the slopes of log *P* vs. log *K_ow_* (or log *P* vs. log *D_ow_*) in the permeability, lipophilicity, and MW correlation analyses. These slopes (coefficient *a*) are significantly smaller than unity for the linear free energy relationship between membrane partitioning and octanol/water partitioning (*K_m_* vs. *K_ow_*, Equation (5)), and this can be attributed to a number of factors. First, the transport rate-limiting barrier of the buccal mucosa can be less lipophilic compared to octanol. This can lead to a slope significantly smaller than unity in the linear free energy relationship between water-to-membrane and water-to-octanol partitioning for buccal membrane permeation. Second, the influences of the polar pathway and underlying tissues of the buccal tissues can create lower and upper boundaries, respectively, for the buccal permeability range to accurately determine the slope (coefficient *a*) in the linear free energy relationship. When the permeability range is narrow in the permeability vs. lipophilicity relationship, the data points at the boundaries or outside this range can “skew” the data, resulting in an apparent slope less than the actual value in the “log *P* vs. log *K_ow_* and *MW*” (or “log *P* vs. log *D_ow_* and *MW*”) correlation. Although employing the exclusion criteria of log *K_ow_* (or log *D_ow_*) < −1 and < 0 could minimize the impact of the polar pathway and reduce this “skewing” effect in the data analysis, as shown in the improvement of the correlations in Table 3, the use of these exclusion criteria might not be sufficient. For example, there was no improvement in the correlation after applying the log *D_ow_* < −1 and < 0 exclusion criteria in the correlations in Table 2. Third, the variability of the permeability data in the previous studies and the uncertainties of log *D_ow_* can affect the correlations. Particularly, the general improvement of the “log *P* vs. log *K_ow_* and *MW*” correlations with the pH independent permeants (i.e., without changes in ionization such as fraction of ionization due to pH) over the correlations of “log *P* vs. log *D_ow_* and *MW*” (first three rows in Table 2 vs. Table 3) can be attributed to the decrease in uncertainties related to permeant ionization due to pH. Previous studies have suggested the possibility of membrane pH that is different from solution pH surrounding the biological membrane (e.g., skin, GI) [129,130,131], and this type of phenomena can lead to errors in calculating the fraction of ionization for buccal membrane permeation in the analyses. Regardless of these influencing factors and uncertainties in the present permeability analyses, the results indicate apparent correlation slopes of buccal membrane permeation vs. log *K_ow_* that are significantly smaller than those of other biological membranes such as lipid bilayers (~2.0–2.8), skin (~0.7–0.8), and cornea (~0.5) [31,122,132], except when the model of Equation (6) was used. In addition, there is a lack of molecular size (or MW) dependence for the permeation of small molecules across the lipoidal barrier of the buccal membrane, which may be “masked” by data variability, when all the data were included in the analyses. When the exclusion criteria were applied to the permeants to take into account the model limitations and the uncertainties in permeant pH-ionization and partitioning, a reasonable quantitative structure permeability relationship was observed: log *P* = 0.2 log *K_ow_*−0.0033 MW−4.8 (with R^2^ = 0.615, Table 3 last row).

## 6. Effective Pore Size for the Permeation of Macromolecules

**Figure 6:**
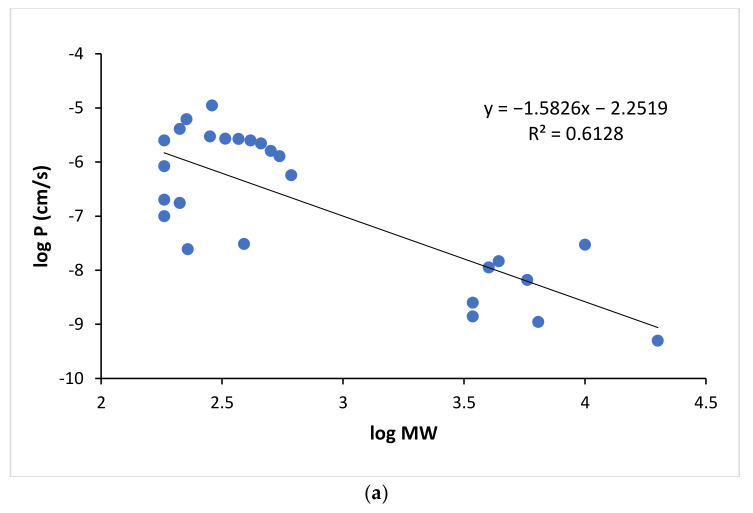

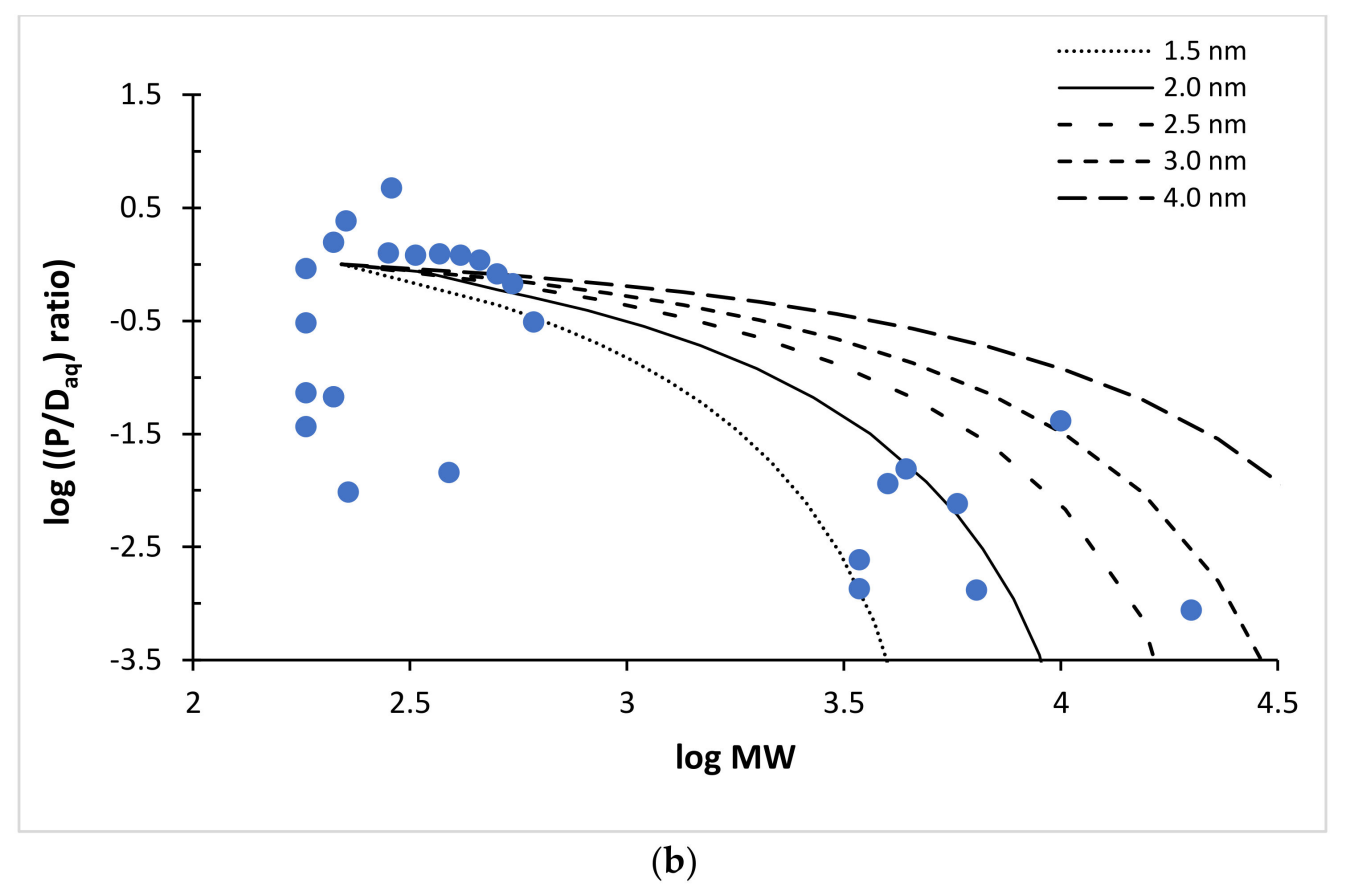
(**a**) Relationship between log *P* and log *MW* of polar permeants (pH independent and log *K_ow_* < −1) and macromolecules from Table A1. (**b**) Comparison of the log (*P*/*D_aq_*) ratio and log *H* ratio vs. log *MW* of the polar permeants and macromolecules. The reference *P*/*D_aq_* and *H* values in (**b**) are those of a hypothetical small permeant with permeability coefficient and MW equivalent to the average values of the permeants with MW < 300 Dalton in (**a**). Symbols: experimental *P*/*D_aq_* ratio data. Lines: *H* ratios calculated with pore radius of 1.5 nm (dotted), 2 nm (solid), 2.5 nm (dash-dot), 3 nm (short dashes), and 4 nm (dashes) using Equation (8).

The permeation of macromolecules across a biological membrane is anticipated to be through the paracellular route of the membrane. This transport pathway can be modeled by the polar pathway and hindered transport theory (Equations (7) and (8)). Figure 6a presents the relationship between the permeability coefficients and MW of the macromolecules and the polar permeants that have log *K_ow_* < −1 and are not pH dependent (i.e., not weak acid or weak base) for the buccal mucosa. With the inclusion of the macromolecules in the permeability analysis, unlike the analysis of only small permeants, a steep size dependence of permeability was observed with a linear regression slope of −1.58 and R^2^ = 0.613 for the log *P* vs. log *MW* relationship.

To examine the effective pore size of the polar pathway, the ratios of the permeability and diffusion coefficients were calculated and compared to a reference (Equation (9)). The reference point in this analysis (Permeant 2 in Equation (9)) was a hypothetical small permeant with an average permeability coefficient and MW of the small permeants that have MW < 300 Dalton in Figure 6a. Figure 6b shows the relationship between the *P*/*D_aq_* ratio and MW from the permeability data and the *H* ratio calculated using Equation (8), based on the reference point. The theoretical hindered transport relationship of molecular size and pore size shows a significant decrease in the *H* ratio when the molecular size increases from 300 to 20,000 Dalton and the pore size (radius) decreases from 4 nm to 1.5 nm. By comparing the theoretical hindered transport *H* ratios and the experimental *P*/*D_aq_* ratios, the result suggests an effective pore radius in the range of 1.5 to 3 nm for the buccal polar pathway (or paracellular pathway). The result in this analysis is in agreement with that in a previous study that investigated the polar pathway using a polymer of different molecular sizes (polyethylene glycol) [96]. In addition, the effective pore size of the buccal mucosa was in the same order of magnitude as other biological membranes such as skin, cornea, conjunctiva, nail, and GI mucosal monolayer [124,126,133,134].

## 7. Permeability Analysis Discussion and Consideration

The present review examined the permeability of porcine buccal mucosa and the major findings are as follows. Large variability of the permeability data was observed in previous studies from different research groups even when the permeability measurements were performed under similar experimental conditions (Figure 1). In general, the correlation between buccal membrane permeability and permeant lipophilicity was relatively poor (Figure 2) and no general permeability-to-MW relationship was observed (Figure 4) when either permeant lipophilicity (as indicated by *K_ow_*) or MW was used as the single independent variable in the analyses of the permeability data for all permeants.

To further investigate the relationship between permeant lipophilicity and the permeability of the lipoidal pathway in the buccal mucosa, the permeability was described better by log *D_ow_* than log *K_ow_* (Figure 3 vs. Figure 2) consistent with the transport theory of uncharged permeants due to pH dependent ionization of the permeants (i.e., weak acid and weak base) when all permeants were used in the analyses. However, the correlation between log *P* and log *D_ow_* was still relatively poor with no improvement in the correlation when permeant MW was incorporated as an additional independent variable in the analyses; there was no observable effect of permeant MW on the permeability. To take into account the contribution of the polar pathway (*P_p_*) to membrane permeability, the permeability data were also evaluated by allowing only permeants with log *D_ow_* > −1 or > 0 in the analyses. These exclusion criteria did not improve the correlations of the “log *P* vs. log *D_ow_* and *MW*” relationship for the permeants studied (Table 2). A hypothesis of the poor correlations is data variability and errors related to the fraction of ionization calculations (e.g., membrane pH different from solution pH and errors introduced in the calculations of log *D_ow_*). The data were therefore analyzed using only pH independent permeants (permeants without fraction of ionization as a function of pH).

For the pH independent permeants, a better correlation was observed in the log *P* vs. log *K_ow_* relationship (Figure 5 vs. Figure 2). The correlation improved with the incorporation of MW as an additional independent variable and further improvement was observed with the exclusion of the permeants that likely utilize the polar pathway, i.e., by limiting the analyses to only permeants with log *K_ow_* > −1 and > 0 (Table 3). When these exclusion criteria were applied, a reasonable quantitative structure permeability relationship of “log *P* vs. log *K_ow_* and *MW*” was observed (R^2^ = 0.615). Another observation in the analyses is the coefficient of log *K_ow_* in the “log *P* vs. log *K_ow_* and *MW*” relationship (coefficient *a* in Equation (11)). This coefficient denotes the slope of the linear free energy relationship between *K_m_* and *K_ow_* (see Equation (5)). The slope of 0.2 is smaller than those observed with other biological membranes and suggests that the barrier domain of buccal membrane permeation is less lipophilic than octanol.

In the investigation of the relationship between permeant MW and the permeability of the polar pathway in the buccal mucosa using the macromolecules and pH independent permeants that have log *K_ow_* < −1, a correlation between log *P* and MW was observed. The result of the hindered transport analysis suggests an effective pore radius of 1.5 to 3 nm in the buccal mucosa for the permeation of polar permeants and macromolecules. This pore size value is in the same order of magnitude as other biological membranes such as skin, cornea, conjunctiva, nail, and GI mucosal monolayer.

It should be pointed out that the present review is focused on porcine buccal mucosa because (a) the majority of the permeability data in previous studies were from this animal model and (b) the similarity between human and porcine buccal mucosae. In addition, the permeability data presented in this review can be different from the permeability for drug delivery in practice because drug formulations usually contain excipients that can enhance membrane permeability for more effective drug delivery and this review is focused only on the intrinsic permeability of the buccal mucosa. The conclusion from the analyses (model of Equation (10)) using all the permeant data collected in this review is affected by (a) data variabilities in the previous studies (i.e., variabilities of *P*, *K_ow_*, and *D_ow_*), (b) model limitations such as the influence of the polar pathway and the difference between *K_m_* and *K_ow_*, and (c) uncertainties in permeant pH-ionization relationship (i.e., the use of *D_ow_*) arise from undefined membrane pH that can be different from the solution pH. The model of Equation (6) could account for the difference between *K_m_* and *K_ow_* but not the other factors. Hence, there was a need to apply certain exclusion criteria in the analyses, leading to a smaller dataset, in order to generate meaningful results. Although a more comprehensive analysis of buccal permeability could not be completed in the present review due to the availability and variability of the data in the literature, the results provide insights into possible quantitative structure permeability relationships of the buccal mucosa.

## 8. Conclusions

The buccal mucosa provides a number of advantages as an alternative route of drug administration. To develop more effective buccal drug delivery systems, knowledge on the intrinsic permeability of the buccal mucosa is essential. However, an extensive review of the permeability data is not available despite the numerous studies and reviews on the topic of buccal drug delivery. In the present review, the intrinsic permeability coefficients of porcine buccal mucosa were collected, a database of the permeability coefficients was generated, and the influences of permeant lipophilicity (as log *K_ow_* and log *D_ow_*) and molecular size (as MW) on the permeability of the buccal mucosa were analyzed. The first observation was the large variability among the published permeability data (of the same permeants). Such variability in buccal permeability studies in the literature could lead to poor correlation (coefficient of determination) in analyzing the permeability data for a possible quantitative structure permeability relationship. In the analyses of all permeability data including the permeants with pH dependent ionization, the permeability was described better by log *D_ow_* than log *K_ow_* for membrane partitioning and permeation, but the correlation was relatively poor with no observable effect of permeant MW. This can be attributed to the difference between solution and membrane pH when using the fraction of unionized permeant in the solution to account for membrane permeation. For the permeability data of pH independent permeants (which are not affected by ionization due to pH in the analysis), a better correlation was observed and the correlation improved with the incorporation of MW as the additional independent variable. The analysis of the relationship between the permeability and partition coefficient of the permeants for the buccal membrane barrier suggested an apparent linear free energy relationship that was less lipophilic than octanol. For the permeability data of macromolecules and polar permeants, an effective pore radius of 1.5 to 3 nm was found for the buccal mucosa using the hindered transport theory. The results obtained in this review could improve our understanding of the buccal mucosal barrier and assist in the development of more effective buccal drug delivery and dosage forms.

## Figures and Tables

**Table 1:** Comparison between the permeability coefficients of porcine buccal tissue and other species and engineered tissues. Data are shown as log *P* (*P* in cm/s).

Permeant	Porcine ^a^	Bovine	Canine	Hamster	Monkey	Human	Engineered Tissue	Ref ^b^
Acebutolol	−7.40, −7.57						−5.46 ^d^	[34]
Alprenolol	−5.70						−4.47 ^d^	[34]
Atenolol	−7.55 to −6.72						−6.41 ^e^, −5.68 ^d^	[34,39]
Caffeine	−5.57 to −4.63			−2.21			−4.96 ^e^, −6.11 ^f^	[39,109,110]
Cathine	−5.50						−5.00 ^e^	[39]
Cathinone	−5.52						−4.92 ^e^	[39]
Estradiol	−4.54, −4.29		−4.88					[111]
Fentanyl	−5.15		−4.54					[112]
Galantamine	−4.93						−4.70 ^d^	[68]
Insulin	−8.18						−5.92 ^d^, −7.10 ^d^	[70,113]
Labetalol	−7.70, −6.26						−5.11 ^d^	[34]
Lidocaine	−6.57 to −4.77			−2.13				[109]
Mannitol	−7.00 to −5.60				−6.51	−6.62, −6.62	−5.39 ^d^, −5.66 ^d^	[34,78,114]
Metoprolol	−8.60 to −4.97					−5.10	−4.47 ^d^	[34,78]
Morphine	−5.88, −5.72	−6.60						[84]
Naltrexone	−5.28, −4.76						−5.07 ^c,f^, −4.98 ^d^	[86,115]
Nicotine	−7.90 to −4.50					−3.77 ^c^		[116]
Norephedrine	−5.67						−5.01 ^e^	[39]
Oxprenolol	−6.05, −5.52						−4.40 ^d^	[34]
Pindolol	−6.92, −6.70						−4.51 ^d^	[34]
Propranolol	−5.89 to −4.85						−5.38 ^d^	[34]
Tertatolol	−6.00						−4.57 ^d^	[34]
Testosterone	−5.96				−4.72	−5.74	−4.70 ^d^	[34]
Timolol	−6.52						−4.62 ^d^	[34]
Verapamil	−4.60			−2.52				[109]
Water	−5.09, −4.94		−4.29		−5.77, −4.62	−6.02	−4.28 ^d^	[34,111,117,118]

^a^ Permeability value from Table A1. ^b^ References listed are for buccal tissues from other species and engineered tissues. References for porcine buccal tissue are listed in Table A1. ^c^ Multiple permeability values are available in the reference and the average value of log *P* was used. ^d^ TR146 cell culture model. ^e^ Caco-2 cell model. ^f^ EpiOral model.

**Table 2:** Regression analysis results of log *P* with log *D_ow_* and *MW* as independent variables (first three rows) and log (*P/D_ow_*) with log *K_ow_* and *MW* as independent variables (last three rows), using the data of all permeants and then permeants with log *D_ow_* > −1 and > 0. The parameters are defined in Equations (6) and (10). The values presented are the least squares means and standard errors of the parameters.

Condition	*a*	*c*	*constant*	R^2^
log *P* vs. log *D_ow_* and *MW*; all data; n = 115	0.29 ± 0.05	−0.0008 ± 0.0009	−5.5 ± 0.3	0.222
log *P* vs. log *D_ow_* and *MW*; log *D_ow_* > −1; n = 88	0.37 ± 0.08	−0.0021 ± 0.0011	−5.3 ± 0.3	0.183
log *P* vs. log *D_ow_* and *MW*; log *D_ow_* > 0; n = 62	0.22 ± 0.10	−0.0007 ± 0.0011	−5.4 ± 0.3	0.072
log (*P/D_ow_*) vs. log *K_ow_* and *MW*; all data; n = 115	0.44 ± 0.05	−0.0014 ± 0.0009	−4.8 ± 0.3	0.588
log (*P/D_ow_*) vs. log *K_ow_* and *MW*; log *D_ow_* > −1; n = 88	0.60 ± 0.09	−0.0031 ± 0.0013	−4.7 ± 0.3	0.382
log (*P/D_ow_*) vs. log *K_ow_* and *MW*; log *D_ow_* > 0; n = 62	0.64 ± 0.12	−0.0027 ± 0.0014	−5.0 ± 0.4	0.279

**Table 3:** Regression analysis results of log *P* with log *K_ow_* and *MW* as independent variables, using the data of pH independent permeants and then with the conditions of log *K_ow_* > −1 and >0. The parameters are defined in Equation (11). The values presented are the least squares means and standard errors of the parameters.

Condition	*a*	*c*	*constant*	R^2^
log *P* vs. log *K_ow_* and *MW*; all data; n = 44	0.16 ± 0.04	−0.0012 ± 0.0007	−5.2 ± 0.2	0.246
log *P* vs. log *K_ow_* and *MW*; log *K_ow_* > −1; n = 28	0.17 ± 0.07	−0.0031 ± 0.0009	−4.8 ± 0.2	0.325
log *P* vs. log *K_ow_* and *MW*; log *K_ow_* > 0; n = 18	0.20 ± 0.06	−0.0033 ± 0.0006	−4.8 ± 0.2	0.615

## Data Availability

Not applicable.

## Appendix A. Permeability Data of Porcine Buccal Mucosa

**Table A1:** Properties of permeants (MW, log *K_ow_*, and pKa), permeability coefficients, and sources of buccal permeation data.

Permeant	MW	log *K_ow_*	pKa	pH	log *D_ow_*	log *P* (cm/s)	Ref
Acebutolol	336.4	1.71	9.46	6.8	−1.56	−7.57	[28]
Acebutolol	336.4	1.71	9.46	7.4	−0.23	−7.40	[34]
Acyclovir	225.2	−1.56	2.52, 9.35	6.8	−1.56	−5.21	[35]
Acyclovir	225.2	−1.56	2.52, 9.35	3.3~8.8	−1.56	−5.20 ^d^	[36]
Alprenolol	249.4	3.1	9.5	7.4	1.18	−5.70	[34]
Amitriptyline	277.4	5.04	9.31	6.8	1.64	−4.89	[27]
Antipyrine	188.2	0.39	1.45	6.8	0.39	−5.65	[37]
Antipyrine	188.2	0.39	1.45	6.8	0.39	−5.27	[27]
Antipyrine	188.2	0.38	1.4	6.8	0.38	−5.67	[29]
Atenolol	266.3	0.22	9.54	7.4	−0.80	−7.30	[34]
Atenolol	266.3	0.22	9.54	7.4	−1.92	−7.39	[38]
Atenolol	266.3	0.22	9.54	6.8	−1.30	−7.55	[27]
Atenolol	266.3	0.22	9.54	6.8	−1.30	−6.88	[28]
Atenolol	266.3	0.22	9.54	7.25	−2.07	−6.72	[39]
Atenolol ^a^	266.3	0.22	9.54	6.8	--- ^c^	--- ^a^	[40]
Benznidazole ^a^	260.2	0.91	--- ^c^	--- ^c^	0.91	--- ^a^	[41]
Bupivacaine	288.4	3.41	8.1	6.8	2.09	−5.10	[37]
Bupivacaine	288.4	3.7	8.1	6.8	2.38	−4.77	[27]
Bupivacaine	288.4	3.41	8.1	6.8	2.09	−4.80	[29]
Buspirone	385.5	2.63	4.12, 7.32	varying pH unionized form data	2.63	−5.54	[42]
Buspirone	385.5	2.63	4.12, 7.32	6.8	2.00	−5.04	[37]
Buspirone	385.5	2.63	4.12, 7.32	6.8	2.80	−4.81	[27]
Buspirone	385.5	2.63	4.12, 7.32	6.8	2.00	−4.76	[29]
Caffeine	194.2	−0.07	10.4	6.8	−0.07	−5.05	[27]
Caffeine	194.2	−0.07	10.4	--- ^c^	−0.07	−4.70	[43]
Caffeine	194.2	−0.07	10.4	6.8	−0.07	−5.49	[37]
Caffeine	194.2	−0.07	10.4	--- ^c^	−0.07	−4.63 ^d^	[44]
Caffeine	194.2	−0.07	10.4	6.8	−0.07	−5.09	[29]
Caffeine	194.2	−0.07	10.4	6.8	−0.07	−4.96	[45]
Caffeine	194.2	−0.07	10.4	7.25	−0.07	−5.57	[39]
Carbamazepine	236.3	2.45	13.9	6.75	2.45	−5.09	[46]
Carprofen	273.7	3.8	4.4	7.4	0.80	−2.63	[47]
Carvedilol ^a^	406.5	4.19	7.8	7.4	3.64	--- ^a^	[48]
Carvedilol	406.5	4.19	7.8	7.4	3.64	−5.24	[49]
Carvedilol ^a^	406.5	4.19	7.8	7.4	3.64	--- ^a^	[50]
Cathine	151.2	0.83	9.2	7.25	−1.12	−5.50	[39]
Cathinone	149.2	1.38	9.2	7.25	−0.57	−5.52	[39]
Decitabine	228.2	−1.89	--- ^c^	7.0	−1.89	−7.64 ^d^	[51]
Diazepam	284.7	2.82	3.4	6.8	2.82	−5.37	[52]
Diazepam	284.7	2.82	3.4	6.8	2.82	−5.32	[45]
Didanosine	236.2	−0.754	9.13	7.4	−0.754	−5.92 ^d^	[53]
Didanosine	236.2	−0.754	9.13	7.4	−0.754	−4.50	[54]
Didanosine	236.2	−0.754	9.13	7.4	−0.754	−6.19	[55]
Dideoxycytidine	211.2	−1.3	--- ^c^	7.1	−1.3	−5.39	[56]
Dideoxycytidine	211.2	−1.3	--- ^c^	7.4	−1.3	−6.76	[57]
Diltiazem	414.5	2.79	7.7	6.8	1.04	−5.14	[27]
Diltiazem	414.5	2.79	7.7	6.0	1.08	−6.34	[58]
Donepezil	379.5	4.86	8.9	7.4	3.35	−4.45	[59]
Doxepin	279.4	4.29	8.96	4.7	0.03	−6.87	[60]
Endomorphin-1	610.7	--- ^c^	--- ^c^	7.1	--- ^c^	−6.25	[61]
Estradiol	272.4	4.01	--- ^c^	7.4	4.01	−4.29 ^d^	[44]
Estradiol	272.4	4.01	--- ^c^	7.4	4.01	−4.54	[43]
Estradiol ^a^	272.4	4.01	--- ^c^	7.4	4.01	--- ^a^	[62]
Felodipine	384.2	3.86	5.07	7.4	3.86	−5.06	[63]
Fentanyl	336.5	4.05	9	7.0	1.41	−5.15	[64]
FITC (fluorescein isothiocyanate)	389.4	--- ^c^	--- ^c^	--- ^c^	--- ^c^	−7.52	[65]
FITC-dextran	4000	--- ^c^	--- ^c^	--- ^c^	--- ^c^	−7.95	[65]
FITC-dextran	4400	--- ^c^	--- ^c^	6.4	--- ^c^	−7.83	[66]
FITC-dextran	10,000	--- ^c^	--- ^c^	--- ^c^	--- ^c^	−7.53	[65]
FITC-dextran	20,000	--- ^c^	--- ^c^	--- ^c^	--- ^c^	<−9.3 ^e^	[65]
FITC-dextran	40,000	--- ^c^	--- ^c^	--- ^c^	--- ^c^	<−9.3 ^e^	[65]
Flecainide	414.3	3.78	9.3	7.4	0.05	−7.19	[67]
Furosemide	330.7	2.03	4.7	6.8	−1.00	−8.48	[27]
Galantamine	287.35	1.8	7.97	6.8	0.60	−4.93	[68]
Hydrocortisone acetate ^a^	404.5	2.19	--- ^c^	--- ^c^	2.19	--- ^a^	[69]
Insulin	5778	--- ^c^	--- ^c^	7.4	--- ^c^	−8.18	[70]
Irinotecan CPT-11	586.7	3.2	9.3	7.4	0.80	−4.24	[71]
Isoniazid	137.1	−0.7	1.8	6.8	--- ^c^	−6.30	[72]
Ketoprofen	254.3	3.12	4.45	7.4	--- ^c^	−5.45	[73]
Labetalol	328.4	3.09	9.3	7.4	0.52	−7.70	[34]
Labetalol	328.4	3.09	9.3	6.8	−0.03	−6.26	[28]
Lamotrigine	265.1	0.89	5.7	7.4	0.89	−5.19	[74]
Lidocaine	234.3	2.1	7.9	6.8	0.97	−4.77	[27]
Lidocaine	234.3	2.44	7.9	6.0	0.53	−6.30	[58]
Lidocaine	234.3	2.44	7.9	7.0	1.49	−6.57	[75]
Lidocaine ^a^	234.3	2.44	7.9	7.0	--- ^c^	--- ^a^	[76]
Lidocaine	234.3	2.1	7.9	7.4	0.97	−5.73	[73]
Lidocaine	234.3	2.44	7.9	7.0	1.49	−5.41	[77]
Mannitol	182.2	−3.1	--- ^c^	7.4	−3.1	−6.70	[34]
Mannitol	182.2	−3.1	--- ^c^	7.4	−3.1	−6.70	[78]
Mannitol	182.2	−3.1	--- ^c^	--- ^c^	−3.1	−5.60	[79]
Mannitol	182.2	−3.1	--- ^c^	6.8	−3.1	−6.08 ^d^	[80]
Mannitol	182.2	−3.1	--- ^c^	7.4	−3.1	−7.00	[81]
Metoprolol	267.4	1.95	9.56	7.4	−0.07	−5.52	[34]
Metoprolol	267.4	1.95	9.56	7.4	−0.21	−4.97	[78]
Metoprolol	267.4	1.95	9.56	6.8	−0.81	−5.89	[27]
Metoprolol	267.4	1.95	9.56	6.8	−0.56	−8.60	[28]
Metoprolol	267.4	1.95	9.56	7.4, 8.0, 8.5, 9.5 ^f^	−0.21	−5.10, −4.46, −4.30, −4.14 ^f^	[82]
Morphine	285.3	0.89	8.21	6.8	−0.70	−5.72 ^d^	[83]
Morphine	285.3	0.89	8.21	--- ^c^	−0.70	−5.88	[84]
Naltrexone	341.4	1.92	8.1	6.8	0.60	−4.76	[85]
Naltrexone	341.4	1.92	8.1	6.8	0.60	−5.28	[86]
Naproxen	230.3	3.18	4.2	6.8	0.58	−5.42	[27]
Nicotine	162.2	1.17	3.04, 7.84	varying pH unionized form data	1.17	−5.00	[87]
Nicotine	162.2	1.17	3.4, 8.2	unionized form data	1.17	−4.88	[88]
Nicotine	162.2	1.3	3.26, 8.06	7.4	0.55	−7.81	[89]
Nicotine	162.2	1.17	3.04, 7.84	4.0	−2.67	−7.90	[58]
Nicotine	162.2	1.17	3.4, 8.2	6.8	−0.25	−5.16	[52]
Nicotine	162.2	1.17	3.4, 8.2	6.8	−0.25	−5.26 ^d^	[79]
Nicotine	162.2	1.17	3.4, 8.2	6.8	−0.25	−5.10	[90]
Nicotine	162.2	1.17	3.4, 8.2	6.8	−0.25	−4.50 ^d^	[80]
Nimesulide	308.3	1.94	6.5	6.8	1.46	−4.52	[27]
Norephedrine	151.2	0.67	9.44	7.25	−1.52	−5.67	[39]
Oligonucleotide	6405	--- ^c^	--- ^c^	--- ^c^	--- ^c^	−8.96	[91]
Omeprazole	345.4	2.23	4.8	7.0	2.23	−5.73	[92]
Ondansetron	293.4	2.4	7.34	unionized form data	2.4	−5.31	[93]
Ondansetron	293.4	2.4	7.34	4.0	−0.94	−6.75	[94]
Oxprenolol	265.4	2.1	9.67	7.4	0.88	−5.52	[34]
Oxprenolol	265.4	2.1	9.67	6.8	0.81	−6.05	[28]
Pindolol	248.3	1.83	9.54	7.4	−0.31	−6.70	[34]
Pindolol	248.3	1.83	9.54	6.8	−0.91	−6.92	[27]
Pioglitazone	356.4	0.425	5.19	7.4	0.42	−5.18	[63]
Pioglitazone	356.4	0.425	5.19	--- ^c^	0.42	−6.15	[95]
Polyethylene glycol	282	−2	--- ^c^	7.4	−2.0	−5.53	[96]
Polyethylene glycol	326	−2.1	--- ^c^	7.4	−2.1	−5.57	[96]
Polyethylene glycol	370	−2.15	--- ^c^	7.4	−2.15	−5.58	[96]
Polyethylene glycol	414	−2.2	--- ^c^	7.4	−2.2	−5.60	[96]
Polyethylene glycol	458	−2.2	--- ^c^	7.4	−2.2	−5.66	[96]
Polyethylene glycol	502	−2.25	--- ^c^	7.4	−2.25	−5.80	[96]
Polyethylene glycol	546	−2.3	--- ^c^	7.4	−2.3	−5.89	[96]
Prilocaine ^a^	220.3	2.11	7.89	7.4	--- ^c^	--- ^a^	[76]
Propranolol	259.3	3.48	9.45	7.4	1.28	−5.89	[34]
Propranolol	259.3	3.48	9.45	6.8	0.83	−4.85	[27]
Propranolol	259.3	3.48	9.45	6.8	1.20	−5.80	[28]
Propranolol	259.3	3.48	9.45	7.4	1.43	−5.64	[97]
Salbutamol ^a^	239.3	1.4	10.3	7.1	−1.80	--- ^a^	[98]
Salmon calcitonin	3431.9	--- ^c^	--- ^c^	4.0	--- ^c^	−8.60	[99]
Salmon calcitonin	3431.9	--- ^c^	--- ^c^	7.4	--- ^c^	−8.86	[100]
Saquinavir	670.8	4.7	7.1	7.4	4.52	−4.26	[101]
Sotalol	272.4	0.24	9.8, 8.3	7.4	−2.16	−7.02	[67]
Steroidal glycoside P57 (extract) ^b^	--- ^c^	--- ^c^	--- ^c^	7.0	--- ^c^	−4.66 ^b^	[102]
Tacrine ^a^	198.3	2.71	9.95	7.4	0.31	--- ^a^	[103]
Tenofovir	287.2	−1.6	--- ^c^	--- ^c^	−1.6	−4.95	[54]
Tertatolol	295.4	2.09	9.8	7.4	−0.37	−6.00	[34]
Testosterone	288.4	3.32	--- ^c^	7.4	3.32	−5.96	[34]
Tetramethylpyrazine	136.2	1.28	--- ^c^	--- ^c^	1.28	−5.04	[104]
Thiocolchicoside	563.6	0.34	--- ^c^	6.8	0.34	−6.72	[105]
Timolol	316.4	1.83	9.21	7.4	−1.71	−6.52	[34]
Triamcinolone acetonide	434.5	2.53	--- ^c^	7.4	2.53	−5.39	[106]
Triamcinolone acetonide	434.5	2.53	--- ^c^	--- ^c^	2.53	−5.55	[43]
Triamcinolone acetonide	434.5	2.53	--- ^c^	--- ^c^	2.53	−5.60	[107]
Triamcinolone acetonide	434.5	2.53	--- ^c^	6.75	2.53	−5.52	[46]
Verapamil	454.6	3.79	8.9	6.8	1.69	−4.60	[27]
Warfarin	308.3	2.6	4.9	6.8	0.69	−5.80	[27]
Water	18	−1.38	--- ^c^	7.4	−1.38	−5.09	[34]
Water	18	−1.38	--- ^c^	7.4	−1.38	−4.94	[81]

^a^ Permeability data with formulations involving organic solvents or enhancers in the permeation experiments. The data were not used in the present analysis. ^b^ Not sufficient information for use in the present analysis. ^c^ Not applicable or not available. ^d^ Multiple permeability values are available in the reference and the average value of log *p* was used. ^e^ Below detection limit. ^f^ Multiple pH conditions are available.

## Appendix B. Derivation of Equations in Model Analyses

From Equation (2) in “Membrane Transport Theory” using a parallel pathway permeation model of lipoidal and polar pathways [120,121], the permeability coefficient of the membrane (*P*) can be expressed as [32]:

(A1) $$P=P_{l}f_{u}+P_{p}$$

where *f_u_* is the fraction of unionized permeant and subscripts *l* and *p* represent the lipoidal and polar pathways, respectively. The fraction of unionized permeant is related to the octanol/water distribution coefficient (*D_ow_*) and octanol/water partition coefficient (*K_ow_*).

(A2) $$D_{ow}=K_{ow}f_{u}$$

(A3)
$$\mathrm{where}\;f_{u}=1/\left(1+10^{\left(pH-pKa\right)}\right)\;\mathrm{for}\;\mathrm{weak}\;\mathrm{acid}$$

(A4)
$$\mathrm{and}\;f_{u}=1/\left(1+10^{\left(pKa-pH\right)}\right)\;\mathrm{for}\;\mathrm{weak}\;\mathrm{base}$$

Combining Equations (A1) and (A2),

(A5) $$P=P_{l}\left(D_{ow}/K_{ow}\right)+P_{p}$$

Assuming that the microenvironment of the lipid lamellae in the membrane can be mimicked by octanol, membrane partition coefficient can be estimated by *K_ow_* (i.e., *K_m_* = *K_ow_*):

(A6) $$P_{l}=\left(K_{m}D_{m}/h_{m}\right)=\left(K_{ow}D_{m}/h_{m}\right)$$

where *D_m_* is membrane diffusion coefficient, *K_m_* is membrane partition coefficient, and *h_m_* is effective thickness of the membrane. The relationship between the permeability of lipid membrane (e.g., lipid bilayer) and permeant lipophilicity defined by its *K_ow_* in Equation (A6) is commonly used in membrane permeation model. Combining Equations (A5) and (A6),

(A7) $$P=\left(D_{ow}D_{m}/h_{m}\right)+P_{p}$$

When the contribution of *P_p_* is minimal, Equation (A7) can be rewritten as:

(A8) $$\log P=\log D_{ow}+\log D_{m}+{h_{m}}^{\prime }$$

where *h_m_′* is a constant related to the effective thickness. The membrane diffusion coefficient can be expressed as [31]:

(A9) $$\log D_{m}=\log D_{0}+c\;MW$$

where *MW* is permeant molecular weight, *D*_0_ is the hypothetical diffusion coefficient of permeants with zero molecular volume, and *c* is a constant. Equation (A8) can be rewritten as:

(A10) $$\log P=\log D_{ow}+c\;MW+constant$$

where *constant* is a constant in the relationship. Or,

(A11) $$\log P=\log K_{ow}+c\;MW+constant$$

when *D_ow_* = *K_ow_* for permeants that are not affected by pH.

A linear free energy relationship can be used to describe the microenvironment of the lipid barrier when it is different from that of octanol for membrane partitioning [122]:

(A12)
$$K_{m}=b{\left(K_{ow}\right)}^{a}$$

Replacing *K_ow_* in Equation A6 by the relationship of Equation (A12) and rearranging the equation as in the derivation from Equations (A5)–(A10),

(A13) $$\log(P/D_{ow})=\left(a-1\right)\log K_{ow}+c\;MW+constant$$
